# Regulation of CD47 expression on CD14^+^ monocytes by interferon-α in PBC patients

**DOI:** 10.3389/fimmu.2023.1256995

**Published:** 2023-12-04

**Authors:** Xi Su, Wenwen Jin, Lizhi Liu, Zifei Zhu, Cunyan Li

**Affiliations:** ^1^ Department of Laboratory Medicine, Hunan Provincial People’s Hospital (the First- Affiliated Hospital of Hunan Normal University), Changsha, China; ^2^ Department of Laboratory Medicine, the First- Affiliated Hospital of Hunan Normal University (Hunan Provincial People’s Hospital), Changsha, China; ^3^ Research Office of Clinical Laboratory, Clinical Translational Medicine Research Institute of Hunan Provincial People’s Hospital (the First-affiliated Hospital of Hunan Normal University), Changsha, China

**Keywords:** PBC, cd47, SIRPα, immune checkpoint, inflammatory response

## Abstract

**Background:**

Primary biliary cholangitis (PBC) is a chronic intrahepatic cholestatic autoimmune liver disease characterized by inflammatory injury of small and medium-sized bile ducts in the liver. The pathogenesis of PBC has yet to be entirely understood. CD47/signal-regulatory protein alpha (SIRPα) is closely related to developing autoimmune diseases by promoting inflammatory response. However, the effect of CD47/SIRPα on inflammatory response in PBC patients is still unclear.

**Objective:**

We investigated the expression of CD47/SIRPα and the effect of inflammatory cytokines on the CD47 expression, analyzed potential autoantibodies against CD47 and the effect of anti-CD47 antibody on the inflammatory response in PBC, provided laboratory basis for the study of the pathogenesis and targets for non-invasive diagnosis and treatment on PBC.

**Methods:**

The expression levels of CD47 and SIRPα on peripheral blood mononuclear cells (PBMC) were measured in 14 patients with PBC (the PBC group) and 13 healthy subjects (the Control group) by flow cytometry (FCM). The PBMC derived from healthy subjects were stimulated with healthy subjects’ serum, PBC patients’ serum, IFN-α or TNF-α, and the CD47 expression level on CD14^+^ monocytes was detected by FCM. The level of serum anti-CD47 antibody or IFN-α in PBC patients and healthy subjects was analyzed by ELISA. FCM was used to examine the TNF-α expression level in CD14^+^ monocytes of healthy subjects stimulated with isotype control antibody, anti-CD47 antibody, LPS or LPS combined with CD47 antibody.

**Results:**

The CD47 expression level on the CD14^+^ monocytes in PBC patients was statistically higher than that in the Control group (*P*<0.01). Compared with the Control group (PBMC+healthy serum), the CD47 expression on CD14^+^ monocyte stimulated with the PBC patients’ serum (PBMC+PBC patients’ serum) was increased (*P*<0.001); the CD47 expression on CD14^+^ monocyte stimulated with IFN-α (PBMC + IFN-α) increased gradually with the increased concentration of IFN-α (*P*<0.05). However, there was no similar trend on CD14^+^ monocyte stimulated with the TNF-α (PBMC+TNF-α) (*P*>0.05). The levels of serum anti-CD47 antibody and IFN-α in the PBC patients were higher than those in healthy subjects (*P*<0.05). The TNF-α expression level in CD14^+^ monocyte stimulated with the LPS (PBMC+LPS) or anti-CD47 antibody+LPS group (PBMC+LPS+anti-CD47 antibody) was significantly increased than that in the Control group (PBMC+isotype control antibody) (*P*<0.01 and *P*<0.001, respectively). The TNF-α expression level in CD14^+^ monocyte stimulated with the anti-CD47 antibody + LPS was higher than that with the LPS (*P*< 0.05).

**Conclusion:**

The CD47 may be related to the pathogenesis of PBC by inflammatory response. The CD47/SIRPα signal were imbalanced in PBC patients. The presence of serum anti-CD47 antibodies in PBC patients provides a laboratory basis for clinical diagnosis and treatment.

## 1 Introduction

Primary biliary cholangitis (PBC) is a chronic intrahepatic cholestatic autoimmune liver disease characterized by inflammatory injury of small and medium-sized bile ducts in the liver. It occurs mainly in middle-aged and older women; the onset is mostly hidden. The early symptoms of the disease are not specific, and it is easy to miss or misdiagnose (1). The pathogenesis of PBC needs to be clarified. The interaction among environmental, genetic, and hormonal factors led to congenital and adaptive immune disorders and loss of self-tolerance (2), leading to antibody and T cell-mediated specific immune attacks against the liver, progressive inflammatory necrosis, and fibrosis of the liver (3, 4). The activation of inflammatory mediators such as type I interferon (IFN-I) and tumor necrosis factor (TNF), innate liver lymphocytes, and natural killer T cells played a crucial role in the pathogenesis of the disease (5). In addition, due to the existence of the ‘gut-liver axis’, when the intestinal mucosa was damaged, Lipopolysaccharides (LPS), the cell wall component of Gram-negative bacteria, entered the liver through the portal vein, which would cause activation of Toll-like receptors 4 (TLR4) and nuclear factor kappa-B (NF-κB) signaling pathway, increased the expression of inflammatory factors, and aggravated the liver from autoimmune damage (6).

CD47 is a transmembrane protein expressed in different cell types, such as thymocytes, T and B cells, monocytes, erythrocytes and nerve cells. It belongs to the immunoglobulin superfamily (7) and is a supramolecular complex composed of integrin, G protein, and cholesterol (8). It interacts with corresponding ligands and mediates cell proliferation, migration, phagocytosis, apoptosis, immune homeostasis, and inhibition of nitric oxide signaling (9, 10). signal-regulatory protein alpha (SIRPα) binds to CD47 to initiate an inhibitory signaling pathway, resulting in weakened phagocytosis of macrophages to malignant cells (11).

The binding of CD47 on erythrocyte to the SIRPα on macrophages prevented erythrocyte phagocytosis by suppressing phagocytic activity. The polycythemia phenotype was restored by intercepting CD47-SIRPα through either anti-CD47 treatment or loss of the inhibitory SIRPα-signal in a PV mouse model (12). Both solid and hematologic malignancies expressed higher levels of CD47, which bound with SIRPα to protect the tumor cell against macrophage-mediated phagocytosis (13–15). The CD47 enabled cancer cells to escape innate and adaptive immune surveillance leading to metastatic spread, which could be restricted by the administration of anti-CD47 antibodies through affecting tumor growth and tumor microenvironment signaling (16, 17). Anti CD47 therapy promoted T cell secretion of pro-inflammatory cytokines in an undifferentiated pleiomorphic sarcoma which expressed highly CD47 (18).

CD47-SIRP α signaling pathway is related to the development of autoimmune diseases. CD47 deficiency improved ocular autoimmune inflammation (19) and furthered macrophage-mediated phagocytosis in type I diabetes (20). CD47 facilitated autoimmune valvular carditis through damaging macrophage efferocytosis and increasing cytokine production (21). T cell activation was regulated by macrophages through CD47/SIRPA in inflammatory bowel disease (22). Jin et al. (7) reported that the CD47 expression level on monocytes in SLE patients was significantly increased, promoting SLE patients’ inflammatory response. However, the effect of CD47/SIRPα on the inflammatory response in PBC patients has not been reported.

Therefore, the present research clarified the expression of CD47/SIRPα on mononuclear cells in PBC patients, investigated the effect of serum inflammatory cytokines on the CD47 expression in PBC patients, analyzed potential autoantibodies against CD47 in PBC patients, and explored the role of CD47 and anti-CD47 antibody in the inflammatory response of PBC, provided laboratory basis for the study of the pathogenesis and targets for non-invasive diagnosis and treatment on PBC.

## 2 Materials and methods

### 2.1 Research object

From December 2021 to February 2023, 14 PBC patients and 13 healthy subjects were selected from Hunan Provincial People’s Hospital (the First Affiliated Hospital of Hunan Normal University). Diagnosis of PBC was based on the published American Association for the Study of Liver Diseases (AASLD) criteria, APASL clinical practice guidance: the diagnosis and management of patients with primary biliary cholangitis (2022) (23). Patients were excluded with one or more of the following conditions: (1) severe cardiovascular disease, kidney disease and other serious diseases; (2) acute and chronic infectious diseases and various non-PBC immune system diseases; (3) pregnant or lactating women; (4) liver cancer or other types of malignant tumor. The ethics committee of Hunan Provincial People’s Hospital approved the exemption of informed consent, because theresearch used the remaining samples after clinical testing, which did not cause additional harm to patients.

### 2.2 Peripheral blood experiments

#### 2.2.1 Extraction of peripheral blood mononuclear cells

EDTA anticoagulant venous blood of PBC patients or healthy subjects diluted 2-4 times with PBS was mixed with Ficoll lymphatic separation solution at 1:1, centrifuged at 400 g, 18°C-20°C for 40 min. The PBMC layer was taken and centrifuged twice to leave the PBMC for use.

#### 2.2.2 Detection of CD47/SIRPα expression on the mononuclear cells

**Figure 1:**
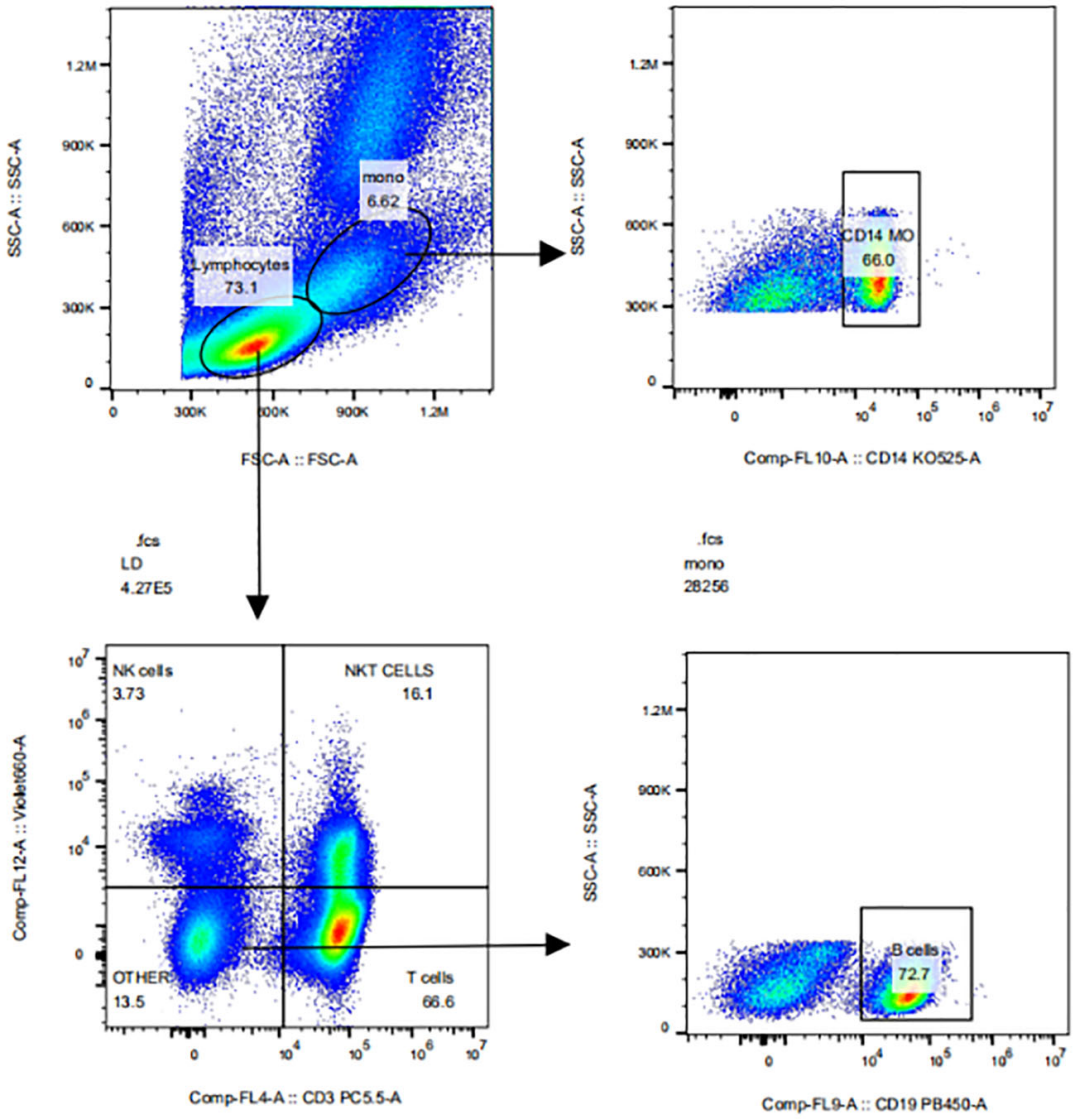
The gate-drawing strategies of CD3+T cells, CD19+B cells, CD56+NK cells and CD14+ monocytes.

After treated with Fixable Viability Dye eF780(#65-0865, eBioscience, USA) and FC receptor blockers (#564765, BD Biosciences, Germany), PBMCs (10^5^ cells/mL) were incubated for 30 min at 4°C in the dark with 5ul of fluorescent dyes CD3-Percp-Cy5.5 (#560835, BD Biosciences), CD19-BV421 (#562440, BD Biosciences), CD14-BV510 (#563079, BD Biosciences), CD56-BV650(#564057, BD Biosciences), SIRPα-APC (#17-1729-42, eBioscience), 20ul of CD16-PE (#555407, BD Biosciences) or CD47-FITC (#556045, BD Biosciences). After washing, the cells were detected by CytoFlex V5-B5-R3 Flow cytometer (No: 38385, Beckman USA). The expressions of CD47 and SIRPα in CD3^+^ T cells, CD19^+^ B cells, CD56^+^ NK cells or CD14^+^ monocytes in PBMC were analysed using flowjo 6.2 software. The gate-drawing strategies for CD3^+^ T cells, CD19^+^ B cells, CD56^+^ NK cells and CD14^+^ monocytes were shown in **Figure 1**.

#### 2.2.3 Detection of anti-CD47 antibody level and IFN-α level

According to the instructions, the serum anti-CD47 level and the IFN-α level in the PBC patients and healthy subjects were detected by ELISA kits (#F-111548-A, FANKEW, China) (#F10665-A, FANKEW, China).

#### 2.2.4 Detection of ANA and AMA

Antinuclear antibody (ANA) and antimitochondrial antibody (AMA) were detected by indirect immunofluorescence method [EUROIMMUN Medical Diagnostics (China) Co., Ltd.], autoimmune liver disease antibodies were detected by membrane strip immunoblotting (Suzhou Haobo Co., Ltd.), and serum immunoglobulins by rate scattering turbidimetric assay (Siemens, USA), liver function was detected by the automatic biochemical analyzer (Hitachi Medical Instrument Co., Ltd.).

### 2.3 *In vitro* experiments

#### 2.3.1 Detection of CD47 expression on the CD14^+^ monocytes

PBMCs were extracted from healthy subjects. PBMC samples were divided into the Control, PBC, IFN-α, and TNF-α groups. Cells were cultured in RPMI-1640 medium containing 10% fetal bovine serum (FBS). According to the grouping, 100 uL of healthy human serum, 100 uL of PBC patient serum, IFN-α (#ab200262, absin, China) (0/10/50) ng/mL, or TNF-α (#ab259410, absin) (0/10/50) ng/mL was added to PBMC (10^5^ cells/mL), mixed and incubated at 37°C, 5% CO_2_ incubator overnight (12 h). Cells were collected and treated with Fixable Viability Dye eF780(#65-0865, eBioscience, USA) and FC receptor blockers (#564765, BD Biosciences, Germany). After washing, 5 μL of fluorescent dye CD14-BV510 (#563079, BD Biosciences) and 20ul of CD47-FITC (#556045, BD Biosciences) were added and incubated at 4°C in the dark for 30 min. After washing, the cells were detected by CytoFlex V5-B5-R3 Flow cytometer (No: 38385, Beckman USA) Flow cytometer.

#### 2.3.2 Detection of TNF-α level in the CD14^+^ monocytes

PBMCs were extracted from healthy subjects. PBMC samples were divided into the CD47 isotype control group, anti-CD47 antibody group, LPS group, and anti-CD47 antibody + LPS group. The cells were cultured in RPMI-1640 medium containing 10% FBS. According to the grouping, 1 μg/mL of isotype control (#14-4321-85, eBioscience), 1 μg/mL of anti-CD47 antibody (#16-0471-81, eBioscience), 3 μg/mL of LPS (#abs47014848, absin), 1 μg/mL of anti-CD47 antibody (#16-0471-81, eBioscience) + 3 μg/mL of LPS (#abs47014848, absin) was added to PBMC (10^5^ cells/mL), respectively. After mixing, they were cultured in 37°C, 5% CO_2_ incubator for 5 h. Cells were collected and treated with Fixable Viability Dye eF780(#65-0865, eBioscience, USA) and FC receptor blockers (#564765, BD Biosciences, Germany). After washing, 5 μL of fluorescent dye CD14-BV510 (#563079, BD Biosciences) was added and incubated at 4°C for 30 min in the dark. Washing, an intracellular staining antibody (TNF-α-PE-c) (#557647, BD Biosciences) was added. Washing again, the cells were detected by CytoFlex V5-B5-R3 Flow cytometer (No: 38385, Beckman USA) Flow cytometer.

### 2.4 Statistical analysis

Statistical analysis was performed using Graph Prism 8.3.0 and SPSS Statistics 25 software. Flow data were analysed using the flowjo 6.2 software. The measurement data of normal distribution were expressed as mean ± standard deviation. One-way ANOVA was used to compare the differences among multiple groups, and *Post Hoc post hoc* test was used for pairwise comparison. The difference between the two groups was analyzed by independent sample t-test or paired t-test. Non-normal distribution data were expressed as median (quartile). Mann-Whitney U test was used for comparison between two groups and multiple groups. *P*<0.05 indicated a statistically significant difference.

## 3 Results

### 3.1 Clinical indicators of patients

A total of 14 patients with PBC and 13 healthy subjects were collected. Among the enrolled 14 PBC patients, 12 cases were positive for ANA, 11 cases were positive for AMA, 1 case was positive for anti-soluble liver antigen/hepatopancreatic antigen antibody (SLA/LP), 1 case for anti-smooth muscle antibody (SMA) and 1 case for anti-hepatocyte cytoplasmic type 1 antibody (LC-1). There was no significant difference in gender and immunoglobulin G between the two groups (*P*>0.05). The levels of alanine aminotransferase (ALT), aspartate aminotransferase (AST), total bilirubin (TBIL), direct bilirubin (DBIL), immunoglobulin M (IgM) and immunoglobulin A (IgA) in PBC patients were higher than those in the control group (*P*<0.05). In contrast, complement 3 (C3) and complement 4 (C4) were opposite (*P*<0.05). The clinical indicators of PBC patients and healthy subjects enrolled in this research were shown in **Table 1**.

**Table 1:** Clinical indicators of the study subject.

	patients with PBC (n=14)	Control (n=13)	*P*-value
age (years) Female (n, %) ast (U/L) alt (U/L) alp (U/L) ggt (U/L) TBIL (umol/L) DBIL (umol/L) γ-globulin IgG (g/L) igA (g/L) igM (g/L) C3 (g/L) C4 (g/L) ANA (n, %) *SLA/LP* (n, %) sma (n, %) *LKM-1* (n, %) *LC-1* (n, %) ama (n, %)	62.00±10.72 13(92.86%) 56.25(42.53, 92.83) 44.85 (17.03, 92.88) 149.66±84.03 111.60(26.80, 245.95) 37.83(21.79, 57.28) 20.09(9.07, 48.68) 29.57±10.48 19.57±9.67 3.41(2.59, 4.58) 2.07(1.52, 6.16) 0.74±0.28 0.14±0.09 12(100%)a 1(7.14%) 1(7.14%) 0 1(7.14%) 11(78.57%)	50.77±7.73 11(84.62%) 22.90(21.45, 26.40) 22.60(15.15, 28.10) - - 12.57(10.51, 14.26) 3.06(2.13, 3.31) - 12.71±2.01 2.08(1.74, 2.99) 0.97(0.82, 1.64) 1.11±0.15 0.26±0.08 - - - - - -	0.005 0.496 <0.001 0.033 - - 0.001 <0.001 - 0.053 0.011 0.024 <0.001 0.002 - - - - - -

-, No data; ^a^ Data were collected from only 12 patients with PBC. AST: aspartate aminotransferase, ALT, alanine aminotransferase; ALP, alkaline phosphatase; GGT, glutamyl transpeptidase; TBIL, total bilirubin; DBIL, direct bilirubin; IgG, immunoglobulin G; IgA, immunoglobulin A; IgM, immunoglobulin M; C3, complement 3; C4, complement 4; ANA, Antinuclear antibody; SLA/LP, anti-soluble liver antigen/hepatopancreatic antigen antibody; SMA, anti-smooth muscle antibody; LKM-1, anti-liver kidney microsome type 1 antibody; LC-1, anti-hepatic cytosolic antigen type 1 antibody; AMA, anti-mitochondrial antibody.

### 3.2 The expression levels of CD47 and SIRPα on mononuclear cells in PBC patients

**Figure 2:**
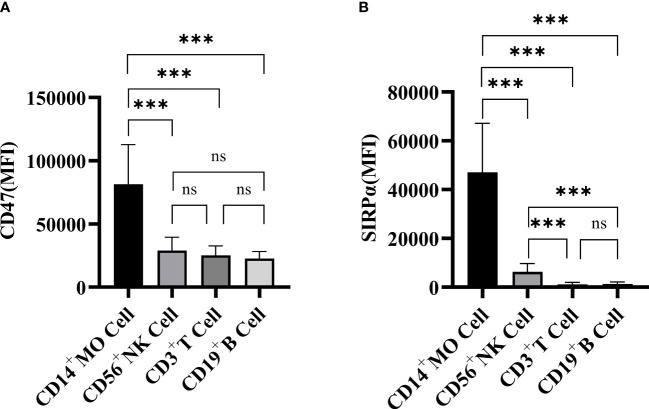
The expression of CD47 **(A)** and SIRPα **(B)** on CD3^+^ T cells, CD19^+^ B cells, CD56^+^ NK cells and CD14^+^ monocytes in PBC patients. ns: no statistical significance; ***P< 0.001, **P< 0.01, *P< 0.05.

The mean fluorescence intensity (MFI) of CD47 and SIRPα expression on the PBMC surface of PBC patients was shown in **Figure 2**. The expression levels of CD47 on CD14^+^ monocytes (81484 ± 31179), CD56^+^ NK cells (28982 ± 10467), CD3^+^ T cells (25124 ± 7565) and CD19^+^ B cells (22639 ± 5596) in PBC patients were statistically different (F=24.705, *P*<0.001). The CD47 expression level on CD14^+^ monocytes was higher than that on CD56^+^ NK cells, CD3^+^ T cells or CD19^+^ B cells in PBC patients (t=5.812, 6.573, 6.951, *P*<0.001, respectively), there were no significant difference in the CD47 expression levels among the CD3^+^ T cells, CD19^+^ B cells and CD56^+^ NK cells in PBC patients (*P*>0.05) (**Figure 2A**).

The SIRPα expression levels on CD14^+^ monocytes (47112 ± 20060), CD56^+^ NK cells (6332 ± 3348), CD3^+^ T cells (1166 ± 785) and CD19^+^ B cells (1308 ± 813) in PBC patients were statistically different (F=33.367, *P*<0.001). The SIRPα expression level on CD14^+^ monocytes was higher than that on CD56^+^ NK cells, CD3^+^ T cells or CD19^+^ B cells in PBC patients (**Figure 2B**).

**Figure 3:**
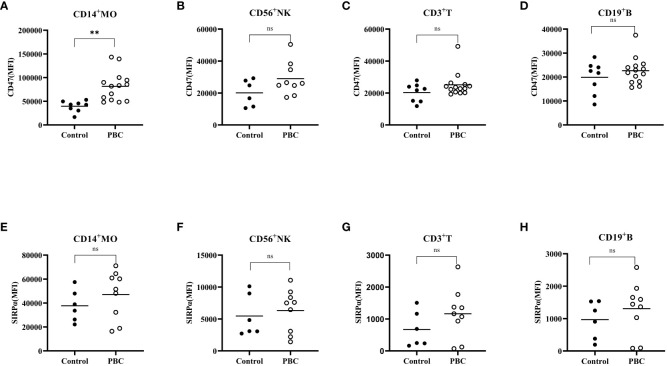
The expression of CD47 **(A–D)** and SIRPα **(E–H)** on CD3^+^ T cells, CD19^+^ B cells, CD56^+^ NK cells, and CD14^+^ monocytes in the Control group and PBC group. ns: There was no statistical significance between the two groups; ***P< 0.001, **P< 0.01, *P< 0.05.

The CD47 expression level (**Figures 3A–D**) on CD14^+^ monocytes in PBC patients was higher than that in the Control group (81484 ± 31179, 39537 ± 11773; t=3.628, *P*<0.05) (**Figure 3A**). There was no significant difference in the SIRPα expression levels on CD3^+^ T cells, CD19^+^ B cells, CD56^+^ NK cells or CD14^+^ monocytes between PBC patients and the Control group (*P*>0.05) (**Figures 3E–H**).

### 3.3 Serum IFN-α expression in PBC patients

**Figure 4:**
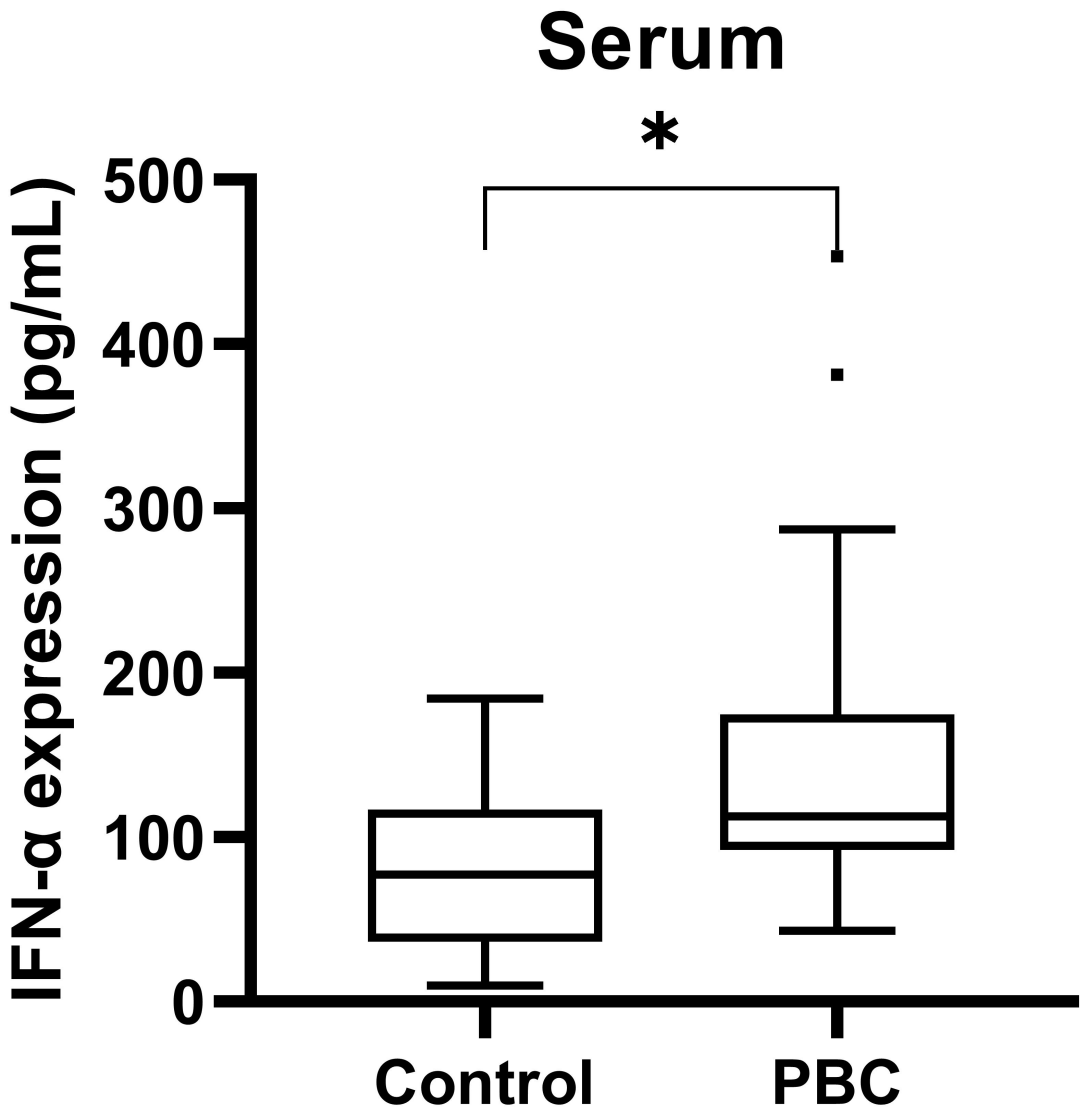
The serum IFN-α level in PBC patients and healthy examiners. ***P < 0.001, **P < 0.01, *P < 0.05.

The serum IFN-α level in PBC patients and healthy examiners were measured by ELISA. PBC patients had higher serum IFN-α levels than that in healthy examiners [(112.57(92.23, 174.78) pg/mL, 77.20(36.51, 116.70) pg/mL; Z=-2.10, *P*<0.05, **Figure 4**)].

### 3.4 The CD47 expression level on CD14^+^ monocytes incubated with PBC patients serum or recombinant IFN-α

**Figure 5:**
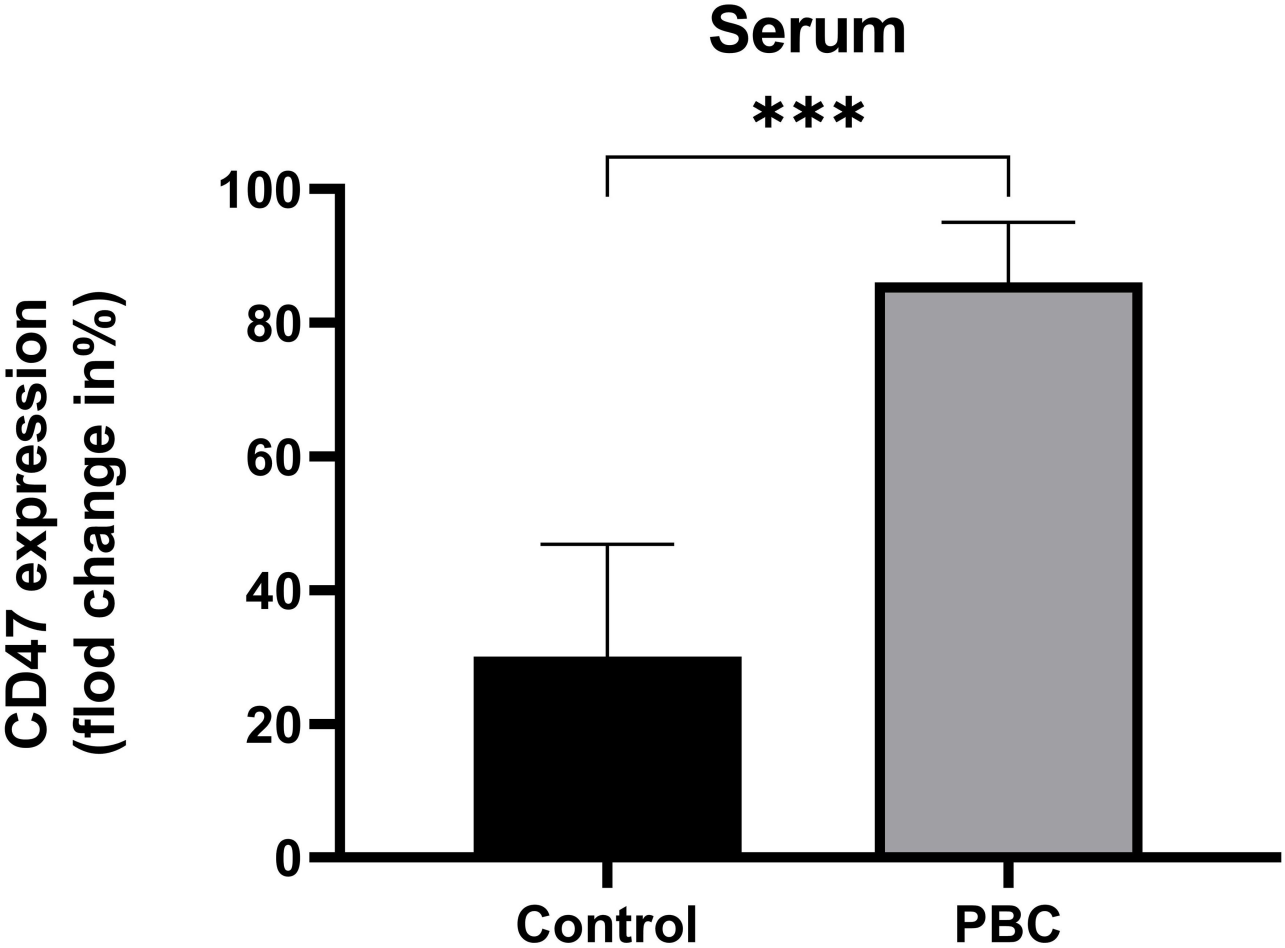
The CD47 expression levels on CD14^+^ monocytes in the Control group and PBC group. ***P ≤ 0.001, **P< 0.01, *P< 0.05.

PBMCs of healthy subjects were incubated with healthy subjects serum (n=5), PBC patients serum (n=9), different concentrations of recombinant IFN-α (n=4) or recombinant TNF-α (n=4), and the CD47 expression levels on cultured cells were detected. The CD47 expression level on CD14^+^ monocytes in the PBC group was significantly higher than that in the Control group (86.06% ± 8.98%, 30.09% ± 16.75%; t=6.94, *P*=0.001, **Figure 5**).

**Figure 6:**
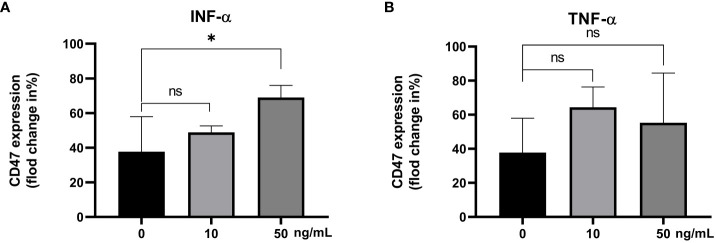
The CD47 expression levels on CD14^+^ monocytes incubated with different concentrations of the IFN-α **(A)** and TNF-α **(B)**. ns: There was no statistical significance between the two groups; ***P< 0.001, **P< 0.01, *P< 0.05.

The CD47 expression levels in the IFN-α 0 ng/mL group, IFN-α 10 ng/mL group and IFN-α 50 ng/mL group were statistically different (F=6.41, *P*<0.05). The CD47 expression level in IFN-α 50 ng/mL group was higher than that in IFN-α 0 ng/mL group (69.03% ± 6.97%, 37.75% ± 20.21%; t=2.93, *P*=0.05, **Figure 6A**). The CD47 expression levels in the different concentrations of recombinant TNF-α groups were not statisticallydifferent (P>0.05), **Figure 6B**.

### 3.5 Serum anti-CD47 antibody level in PBC patients

**Figure 7:**
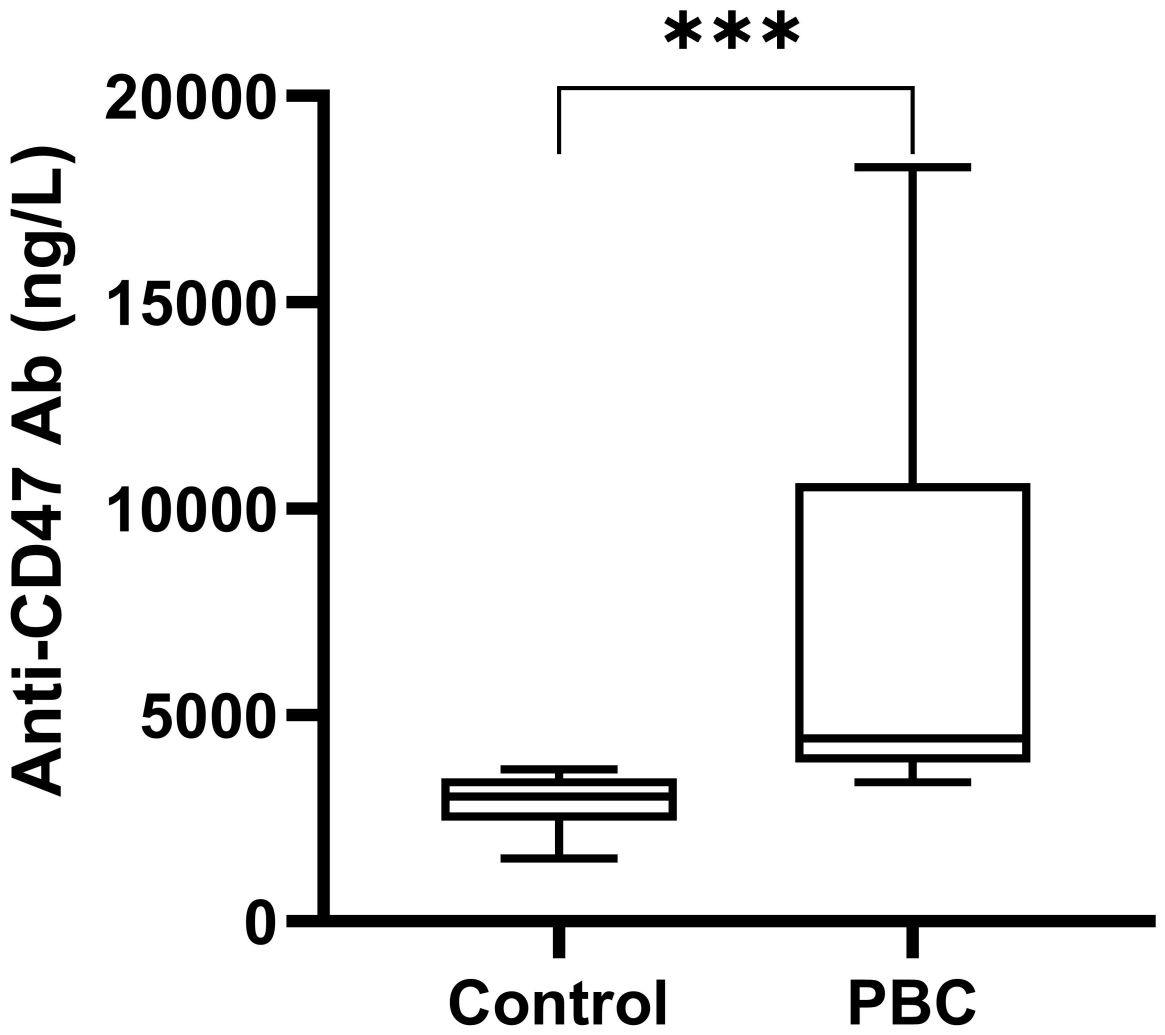
The serum anti-CD47 antibody level in the PBC group and the Control group. ***P≤ 0.001, **P< 0.01, *P< 0.05.

The serum anti-CD47 antibody levels in PBC group (n=10) and the Control group (n=9) were detected by ELISA. The level of serum anti-CD47 antibody in PBC patients was significantly higher than that in the Control group [4437.50(3850.00, 10631.25) ng/L,3018.75(2443.75, 3475.00) ng/L; Z=-3.43, *P*=0.001, **Figure 7**)].

### 3.6 The TNF-α expression level in CD14^+^ monocytes incubated with the anti-CD47 antibody

**Figure 8:**
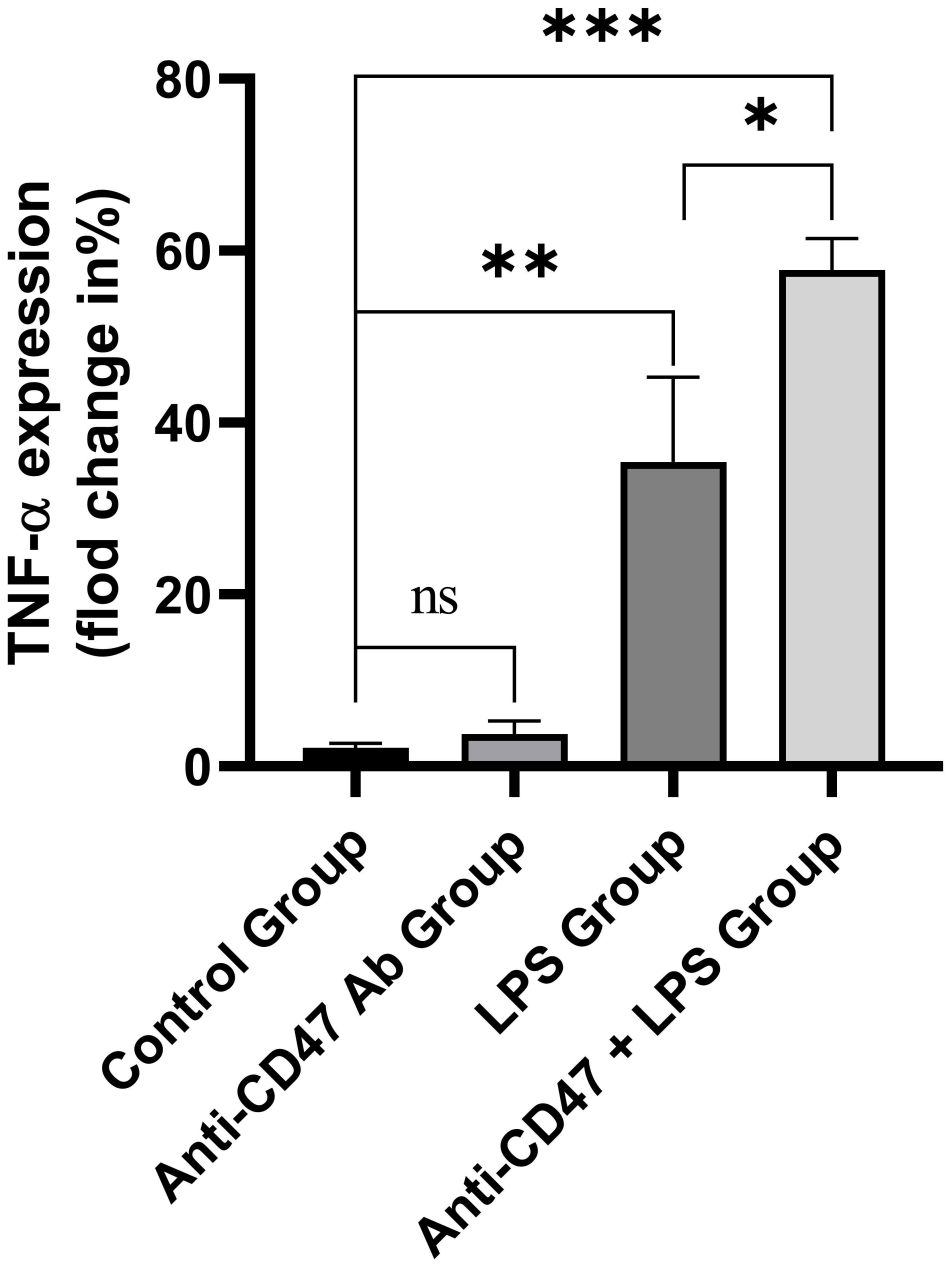
The TNF-α expression levels in CD14^+^ monocytes in the isotype control group, anti-CD47 antibody group, LPS group and anti-CD47 antibody+LPS group. ns: There was no statistical significance between the two groups; ***P< 0.001, **P< 0.01, *P< 0.05.

PBMC in healthy subjects was incubated with an isotype control antibody, anti-CD47 antibody, LPS or anti-CD47 antibody+LPS. The TNF-α expression levels in CD14^+^ monocytes in cultured PBMC were detected. There were significant differences in the TNF-α expression in CD14^+^ monocytes among the isotype control group, anti-CD47 antibody group, LPS group and anti-CD47 antibody + LPS group (F=76.58, *P*<0.001). The TNF-α expression level in CD14^+^ monocytes in the anti-CD47 antibody group was not different from that in the isotype control group (3.76% ± 1.51%, 2.19% ± 0.51%; t=1.70, *P*>0.05), the TNF-α expression level in CD14^+^ monocytes in the LPS group was higher than that in the isotype control group (35.43% ± 9.85%, 2.19% ± 0.51%; t=5.84, *P*<0.01), the TNF-α expression level in CD14^+^ monocytes in the anti-CD47 antibody + LPS group was higher than that in the LPS group (57.80% ± 3.61%, 35.43% ± 9.85%; t=3.70, *P*<0.05) (**Figure 8**).

## 4 Discussion

Immunosurveillance among normal cells, defective cells and foreign pathogens is regulated by cell surface receptors, which mediate the interaction between immune cells and their targets. These ‘ self ‘ signal markers interact with proteins expressed on the surface of phagocytes to inhibit phagocytosis (24). CD47 is a protein expressed in almost all body cells, providing a ‘ do not eat me ’ signal to host phagocytes (including neutrophils and macrophages) (25). Cancer cells escaped the recognition and killing of host phagocytes by expressing high levels of CD47 (26, 27), indicating that pathogenic results will occur in the case of CD47 overexpression. The present research found increased CD47 expression level on CD14^+^ monocytes in PBC patients, consistent with the report (7).

When the CD47 and SIRPα are expressed on the monocytes at the same time, the sum of the two signals may determine the final effect of CD47 and SIRPα on cell response (28). Our research showed that the CD47 expression level was increased in PBC patients. In contrast, the expression of SIRPα was normal on CD14^+^ monocytes in PBC patients, suggesting that immune regulation of the CD47/SIRPα signals were imbalanced in PBC patients. The research also showed IFN-β and IFN-γ/TNF-α decreased erythrophagocytosis by human monocytes *in vitro*, which was independent from the increase in SIRP-α or SHP-1 expression (29).

Our research also showed that the PBC patients’ serum with increased IFN-α level could improve the CD47 expression on CD14^+^ monocytes from healthy subjects. Recombinant IFN-α also increased the CD47 expression on CD14^+^ monocytes in a dose-dependent manner. The data showed enhanced expression of IFN-I and toll-like receptor-3 in PBC (30) and IFN-I signaling as a necessary component of the sex bias in murine autoimmune cholangitis (31). The self-derived IFN inducers and a lack of negative feed-back signals downregulating the IFN response contributed to the continuous IFN production in SLE (32). Therefore, we speculated that increased serum IFN-α caused by self-derived IFN inducers or a lack of negative feed-back signals could promote CD47 expression in CD14^+^ monocytes in PBC patients.

PBC is an autoimmune disease, which can produce a variety of autoantibodies, which helps to diagnose autoimmune diseases. Our research found that the level of anti-CD47 autoantibodies in PBC patients was higher than that in healthy subjects, suggesting that there were autoantibodies against CD47 in PBC patients. Anti-CD47 antibody enhanced the phagocytosis of macrophages by binding to the Fc receptor on the macrophages, which might destroy the interaction of CD47/SIRPα, increase the phagocytic activity of macrophages, lead to an aggravation of experimental autoimmune encephalomyelitis and autoimmune nephritis (33, 34).

Studies have shown that the immune response of effector CD4^+^ T cells (Th1, Th17 and follicular helper T cells) and CD8^+^ T cells to autoantigens (expressed by hepatocytes and biliary epithelium) was related to PBC pathogenesis (35, 36). Activated Th1 and Th17 cells released various inflammatory factors to promote the occurrence and development of PBC (5). When PBC patients were exposed to LPS due to infection, LPS could bind to cell surface receptors (such as TLR 4/CD14) and induced the secretion of pro-inflammatory cytokines (such as TNF-α, IL-1, IL-6, IL-8), promoting inflammation. Our results showed that the anti-CD47 antibody could not promote TNF-α expression in CD14^+^ monocytes. However, LPS could, and TNF-α expression level was higher when stimulated with the combination of the anti-CD47 antibody and LPS. We assumed that anti-CD47 antibody can enhance the pro-inflammatory effect of LPS, aggravate liver lesions, and form a vicious circle.

In summary, increased inflammatory cytokines promote the expression of CD47 on the CD14^+^ monocytes in PBC patients. There was anti-CD47 antibody in PBC patients which could enhance the pro-inflammatory effect of LPS, aggravate liver lesions, and form a vicious circle. Blocking the imbalance of CD47/SIRPα signal may contribute to treatment for PBC patients.

## Data availability statement

The original contributions presented in the study are included in the article/supplementary material. Further inquiries can be directed to the corresponding author.

## Ethics statement

The studies involving humans were approved by The Medical Ethics Committee of Hunan Provincial People’s Hospital. The studies were conducted in accordance with the local legislation and institutional requirements. Written informed consent for participation was not required from the participants or the participants’ legal guardians/next of kin because This research utilized samples obtained after clinical testing without causing harm to patients.

## Author contributions

CL: Conceptualization, Supervision, Writing – review & editing. XS: Writing – original draft, Data curation, Software. WJ: Writing – original draft, Investigation, Methodology. LL: Writing – original draft, Formal Analysis. ZZ: Data curation, Investigation, Resources, Writing – review & editing.
